# Immediate Root Filling

**Published:** 1893-07

**Authors:** Junius E. Cravens

**Affiliations:** Indianapolis.


ARTICLE VI.

### IMMEDIATE ROOT FILLING.

BY JUNIUS E. CRAVENS, D. D. S., INDIANAPOLIS.

**Read before the joint session of the Iowa and Illinois Dental Societies, at Rock Island, May 9, 1893.**

"Is Immediate root-filling advisable: 1st, after heroic extirpation of live pulp? 2nd, after removing devitalized pulp? 3rd, after removing putrescent pulp? 4th, where there is a peridental membrane abscess with fistulous opening? 5th, the same without fistulous opening. 6th, what preparatory treatment should each condition receive, and with what material should roots be filled?" Surely to answer all these interrogatories would require an inspiration. Where is our Isaiah?

To begin with, what is the purpose of filling a canal in a root at all? The canal under consideration is, in a general way, an axial opening extending the length of the root of a tooth. When it contains a living, healthy pulp, practically it is a medullary canal. The pulp itself contains an arrangement of minute blood vessels, nerve fibers and absorbents, supported by connective tissue; in fact, is an organ—or rather, what is left of an organ that has been atrophied almost to extermination, by persistent self-sacrifice. I take it that if a pulp were permitted to finish the task it set out to perform, there would eventually be no canal to vex or make us afraid. And how is this self-sacrificing process arrested?

1st. By development of apical cementum, that so constricts the pulp at that point that its vital power is reduced, and the activity of odontoblasts is arrested. Thus the development of regular dentine is stopped.

2d. By violence; by external irritation; by drugs applied to devitalize the pulp; by death of the pulp from any cause.

3d. By "heroic extirpation of live pulp."

1. In this third proposition we are brought to consider the first of the series of interrogatories under which this subject has been presented by your committee. By "heroic extirpation of live pulp," presumably is meant the forcible dislodgement of the organ, causing a vacation of the canal that already has caused more contention than the Suez Canal, and that will continue to trouble long after De Lesseps' Panama ditch shall have been obliterated and forgotten.

What an awful responsibility your committee has assumed! But a live pulp has been pulled out, and what of it? A minute artery and vein has been snapped in two within or just external to the apical constriction; naturally a slight hemorrhage ensues, sometimes quite flooding the canal, rarely more than this. All hemorrhages have an ending, and blood is easily removed, even from a root-canal. What is to be done? Remove the damning evidence of the blood-curdling tragedy. Dry the canal by any means you may see proper, and without more ado proceed to fill it with whatever material and by whatever method has been most effective in your hands. This done, your duty as a dentist has been performed. I can conceive of no possible beneficent effects to be anticipated from the introduction of medicinal agents into a root-canal, once or twenty times, following extirpation of a live pulp, unless, perhaps, for the checking of persistent hemorrhage—a rare emergency.

To the first interrogatory I will answer, yes; and claim the privilege of explaining that I do not favor the practice of heroic extirpation of live pulps, and consequently do not pursue it.

2. "*Is Immediate root-filling advisable, after removing devitalized pulp?*"

To devitalize a pulp, an application is made to the organ, or in close proximity to it, of some medicinal agent that first arrests circulation within the pulp; stasis is established and death ensues. If removal of the pulp is practiced before putrescence obtains, it must be accomplished by practical extirpation. In this connection I can discern no material difference between a canal that has been vacated by extirpation of a live pulp, and one of extirpation of a devitalized but non-putrescent pulp. To the patient the difference may be very marked, but to the operator practically none.

In the removal of a devitalized but non-putrescent pulp, the same apical hemorrhage may be observed to follow as in extirpation of a live pulp. The separation of the dead from the living within or at the apical constriction may be attended by apparently the same degree of pain in both cases; but the introduction of a broach into and its passage through the devitalized but non-putrescent pulp, while occasing some pain, is far less painful than the penetration of a live pulp to an extent essential to accomplish extirpation.

Usually, I find no occasion for extirpation previous to incipient putrescence, unless there shall present an emergency. This subject, as presented by your committee, does not call for a discussion of emergency measures. My position, then, as to the second interrogatory, is, that the removal of a devitalized pulp that is non-putrescent leaves the canal in precisely the same condition as after extirpation of a live pulp; therefore, my practice would be precisely the same as to the immediate filling of the canal.

3. "*Is Immediate root-filling advisable, after removing putrescent pulp?*"

Now may all saints and phagocytes defend me; for, as I wade, the water appears darker, deep and dangerous. What is a putrescent pulp? In short, a dead pulp in which decomposition has begun. By decomposition there is a watery degeneration of a pulp, and generation of sulphureted hydrogen within the cavity occupied by the pulp.

What effect such degeneration can have upon the surrounding dentine, I am at a loss to discover. Often we observe that pulps have died from some unknown cause and given no sign. Evidently in such obscure cases the usual course of decomposition must have transpired; the complete maceration of tissue, and found the canal empty, even dry and almost inodorous. In such cases the tooth has perhaps retained a natural color; no fistula indicates abscess; all outward appearances indicate a live, healthy pulp. It is useless to speculate upon what becomes of the gas and macerated tissue in such a case. Does it disappear in the surrounding tubuli? If so, why does not discoloration of dentine ensue? But Professor Miller, of Berlin, says in offset that the tubuli retain their original organic contents after death of a pulp, and are not accessible to antiseptic fluids introduced into the canal. If this be true, why should the tubuli be permeable to the fluids resulting from decomposition of the pulp in the canal? And suppose, for the sake of argument, that the water of decomposition may be, has been absorbed by tubuli; what has become of the sulphureted hydrogen also generated in the canal? Does any one suppose that the gas has also been absorbed by the little tubes? And if the sulphureted hydrogen were so absorbed, is there any medicinal or purely chemical agent that can be appealed to for the removal of the gas from dentine? Iodoform may be introduced into a canal and cover up the odor from sulphureted hydrogen, because iodoform can out-stink any gas under the sun.

A platinum wire may be introduced into a canal and heated by electricity; a very effective way to dry the walls of the canal, certainly; but the odor of scorched bone is scarcely preferable to sulphureted hydrogen emanating from a pulp-canal Any solid substance introduced into a canal would displace any gas found there; the platinum wire would do this without being heated, but the odor would remain. The odor of sulphureted hydrogen will cling to a glass tube through which it has been passed. After all, what deleterious effects are to be anticipated from the odor that clings to the walls of a canal from which a putrescent pulp has been removed?

After removal of a putrescent pulp, I would dry the canal and immediately and permanently fill it.

4. "*Is Immediate root-filling advisable, where there is a peridental membrane abscess with fistulous opening?*"

On this point permit me to quote from an essay on "Management of Pulpless Teeth," read by me before the Ninth International Medical Congress, Washington, D. C., 1887. "A fistula may be depended upon to accomplish all necessary drainage of pus from the cavity of an abscess after closure of the apical end of the pulp canal. When no more pus shall be formed, the fistula will disappear for want of exercise." "A fistula is the dentist's friend and best ally; it accomplishes drainage of pus, and thus relieves the pulp-canal of a pernicious service." "A fistula from an alveolar abscess should never be interfered with in any direct manner, but should be encouraged to perform its good offices, and will certainly disappear when its task is done."

In answer to the fourth interrogatory, I hold that immediate root-filling is advisable where there is abscess with fistulous opening.

5. "*Is immediate root-filling advisable, where there is a peridental membrane abscess without fistulous opening?*"

If I correctly comprehend this interrogatory, it refers to what is commonly termed "blind abscess." The darkness of the design of your committee has become truly Egyptian; O for stronger spectacles!

Be kind enough to indulge me in another self-quotation bearing upon this point: "Diagnosis of blind abscess of alveolar process is largely guess-work, because the single item of a wet canal does not prove the presence of pus, nor the existence of an abscess cavity. * * * * When blind abscess exists, the accumulation of pus is so very slight that it will cease altogether after the cause has been removed by closing the apical end of the pulp-canal. In nearly all such cases prompt closure at the apex will be followed by no noticeable inflammation, if proper care be observed in manipulation within the pulp-canal."

In all cases "the apical end of a pulp-canal should be permanently closed as soon as the canal is laudable; that is to say, as soon as free from pus or other fluid, and all obstructive matter." "A clear, clean canal is always laudable."

In reply to interrogatory five, I would remove all obstructive matter from the canal, absorb moisture from it until no more appears, and immediately permanently fill the canal, feeling assured that the operation "will be followed by no noticeable inflammation," if proper care has been observed that nothing has been forced from the canal through the apical constriction.

6. As to the sixth interrogatory, I have given *ad seriatum* my opinion as to "what preparatory treatment should each condition receive," and in closing with consideration of "with what material should roots be filled?" will state that I am a staunch believer in the efficacy of gutta-percha dissolved in chloroform or, what is better, a thin batter of oxychloride of zinc, either to be forced home with gutta-percha points. As a precaution against either preparation mentioned being forced through the opening at the apex of the root, if a canal is fairly penetrable. I first close it at the apical constriction with a slender ribbon of thin tin-foil.

If a canal is too narrow or tortuous for free manipulation, I omit the tin ribbon and trust to careful manipulation to avoid forcing the gutta-percha solution or oxychloride batter through the apical opening. *Beyond the apex danger lurks.*

I hold that immediate root-filling is advisable, and am content to leave measures and materials to men.—*Western Dental Journal.*

---

ARTICLE VII.

